# P037 Recurrent giant cell arteritis as a possible paraneoplastic manifestation of high-grade serous tubo-ovarian carcinoma

**DOI:** 10.1093/rap/rkag113.067

**Published:** 2026-11-03

**Authors:** Rania Ali, Luke Williamson

**Affiliations:** Whipps Cross Hospital, London, United Kingdom; Whipps Cross Hospital, London, United Kingdom

## Abstract

**Introduction:**

Giant cell arteritis (GCA) is the most common systemic vasculitis affecting adults over 50 years of age. Although most cases are idiopathic, an association between vasculitis and malignancy has been recognised, particularly in patients with haematological malignancies. The association between GCA and solid organ malignancies remains uncommon and is poorly understood. We present a case of biopsy-proven GCA with two distinct disease episodes; each temporally associated with the diagnosis and recurrence of high-grade serous tubo-ovarian carcinoma. Notably, the vasculitic symptoms resolved following surgical treatment of the malignancy, raising the possibility of a paraneoplastic phenomenon.

**Case description:**

A 71-year-old woman with biopsy-proven GCA and recurrent high-grade serous tubo-ovarian carcinoma was reviewed in the rheumatology clinic following extensive cytoreductive surgery. She initially presented in 2012 with classical cranial GCA, including temporal headache, scalp tenderness and jaw claudication. Temporal artery biopsy confirmed the diagnosis, and she responded well to high-dose glucocorticoids with subsequent tapering. Several months later, investigations for abdominal symptoms identified a large pelvic mass. In January 2014, she underwent bilateral salpingo-oophorectomy and staging surgery, with histology confirming Stage II bilateral high-grade serous tubo-ovarian carcinoma, followed by adjuvant chemotherapy. Following tumour treatment, her vasculitic symptoms resolved completely, allowing corticosteroid withdrawal, and she remained in remission for almost ten years.

In April 2024, she developed recurrent temporal headache, scalp tenderness and jaw claudication. Temporal artery ultrasound demonstrated bilateral superficial temporal artery mural thickening consistent with recurrent GCA. Prednisolone was commenced with good initial response, and methotrexate was added as a steroid-sparing agent because of osteoporosis risk. However, she remained steroid-dependent, experiencing recurrent symptoms whenever prednisolone was reduced below 6–7 mg daily. Tocilizumab was being considered.

In November 2025, she presented with postmenopausal bleeding. CT imaging demonstrated a 6 cm pelvic mass, and vaginal vault biopsy confirmed recurrent metastatic high-grade serous tubo-ovarian carcinoma. She subsequently underwent extensive cytoreductive surgery in January 2026, including posterior exenteration, bowel resection and end colostomy formation. Methotrexate was discontinued peri-operatively.

Following surgery, she made a good recovery with complete resolution of GCA symptoms, including headache, scalp tenderness and jaw claudication. Given the marked clinical improvement after tumour resection, methotrexate was not restarted, and a gradual prednisolone taper was initiated.

This case highlights the striking temporal relationship between disease activity and malignancy, raising the possibility that recurrent GCA represented a paraneoplastic manifestation of recurrent tubo-ovarian carcinoma rather than isolated primary vasculitis.

**Discussion:**

This case demonstrates a striking temporal relationship between giant cell arteritis activity and the presence of high-grade serous tubo-ovarian carcinoma.

Several observations support a possible paraneoplastic mechanism:

• Initial GCA preceded the diagnosis of ovarian carcinoma.

• Vasculitic symptoms resolved completely following treatment of the primary tumour.

• Nearly ten years later, recurrence of classical cranial GCA symptoms preceded the diagnosis of recurrent ovarian carcinoma.

• The disease became unusually steroid-dependent despite methotrexate therapy.

• Following complete surgical excision of recurrent tumour, GCA symptoms again resolved rapidly, allowing steroid withdrawal.

Paraneoplastic vasculitis has been described in association with solid tumours, although GCA remains an uncommon manifestation. Several mechanisms have been proposed, including tumour-driven immune activation, production of inflammatory cytokines such as IL-6, molecular mimicry and cross-reactive tumour antigens capable of triggering vascular inflammation.

The recurrence of biopsy-confirmed GCA closely paralleling both the initial diagnosis and recurrence of ovarian carcinoma is highly suggestive of an underlying tumour-driven inflammatory process rather than coincidental relapsing GCA.

**Key learning points:**

• Persistent or recurrent GCA should prompt clinicians to consider occult malignancy.

• Recurrence of vasculitic symptoms in patients with a history of cancer may represent a paraneoplastic manifestation rather than isolated autoimmune disease.

• Successful treatment of the underlying malignancy may lead to complete remission of vasculitic symptoms.

• Multidisciplinary collaboration between rheumatology and oncology is essential before commencing biologic immunosuppression in patients with previous malignancy.

• This case adds to the limited literature describing a possible association between giant cell arteritis and high-grade serous tubo-ovarian carcinoma.

• This case describes biopsy-proven GCA occurring before both the primary diagnosis and subsequent recurrence of high-grade serous tubo-ovarian carcinoma. On both occasions, the vasculitis resolved following definitive treatment of the malignancy. The remarkable temporal relationship strongly suggests a paraneoplastic form of giant cell arteritis. Recognition of this association is important, as recurrence of vasculitic symptoms may represent an early clinical marker of tumour recurrence and should prompt consideration of malignancy alongside assessment for vasculitis relapse.

